# Improving ontologies by automatic reasoning and evaluation of logical definitions

**DOI:** 10.1186/1471-2105-12-418

**Published:** 2011-10-27

**Authors:** Sebastian Köhler, Sebastian Bauer, Chris J Mungall, Gabriele Carletti, Cynthia L Smith, Paul Schofield, Georgios V Gkoutos, Peter N Robinson

**Affiliations:** 1 Institute for Medical Genetics and Human Genetics, Charité-Universitätsmedizin Berlin, Augustenburger Platz 1, 13353 Berlin, Germany; 2 Berlin-Brandenburg Center for Regenerative Therapies (BCRT), Charité-Universitätsmedizin Berlin, Augustenburger Platz 1, 13353 Berlin, Germany; 3 Lawrence Berkeley National Laboratory, Mail Stop 64R0121, Berkeley, CA 94720, USA; 4 Dipartimento di Matematica e Informatica, Università di Camerino, Via Madonna delle Carceri 9, 62032 Camerino (MC), Italy; 5 The Jackson Laboratory, Bar Harbor, ME 04609, USA; 6 Department of Physiology, Development, and Neuroscience, University of Cambridge, Cambridge, CB2 3EG, UK; 7 Department of Genetics, University of Cambridge, Downing Street, Cambridge, Cambridge CB2 3EH, UK; 8 Max Planck Institute for Molecular Genetics, Ihnestrasse 73, 14195 Berlin, Germany

## Abstract

**Background:**

Ontologies are widely used to represent knowledge in biomedicine. Systematic approaches for detecting errors and disagreements are needed for large ontologies with hundreds or thousands of terms and semantic relationships. A recent approach of defining terms using logical definitions is now increasingly being adopted as a method for quality control as well as for facilitating interoperability and data integration.

**Results:**

We show how automated reasoning over logical definitions of ontology terms can be used to improve ontology structure. We provide the Java software package *GULO *(Getting an Understanding of LOgical definitions), which allows fast and easy evaluation for any kind of logically decomposed ontology by generating a composite OWL ontology from appropriate subsets of the referenced ontologies and comparing the inferred relationships with the relationships asserted in the target ontology. As a case study we show how to use *GULO *to evaluate the logical definitions that have been developed for the Mammalian Phenotype Ontology (MPO).

**Conclusions:**

Logical definitions of terms from biomedical ontologies represent an important resource for error and disagreement detection. *GULO *gives ontology curators a fast and simple tool for validation of their work.

## Background

The steady increase in biomedical data and publications has led to the need for computational methods for integration and analysis [1]. Controlled vocabularies and ontologies for representing biomedical entities, their terms and their relationships are being developed in order to accomplish this task, with the Gene Ontology [2] (GO) probably being the most successful current bio-ontology. There are multiple ontologies for specific disciplines in biomedicine, which enable scientists to deal with the huge amount of data produced, but a major problem is the lack of interoperability between ontologies of different domains of biomedical knowledge.

The Open Biological and Biomedical Ontologies (OBO) Foundry aims to coordinate a family of ontologies that are interoperable and logically well-formed [3]. These ontologies are constantly growing as knowledge grows (e.g., GO currently comprises over 35,000 classes and over 60,000 relationships), which implies that new quality checking approaches are needed, since manual creation and maintenance of large ontologies is time-consuming and error-prone.

In principle, a good way to develop ontologies is to define concepts in terms of other more elementary (atomic) concepts (building blocks). Groups involved in the GO [4], the Mammalian Phenotype Ontology (MPO) [5], the Human Phenotype Ontology (HPO) [6-8], and the Worm Phenotype Ontology [9] are now developing logical definitions for ontology terms using terms from other ontologies, with PATO, an ontology of phenotypic qualities, being a key tool in this effort [10-14]. For instance, consider the following logical definition of the HPO term *Hypoglycemia*, specified in OBO Format:

[Term]

id: HP:0001943 ! Hypoglycemia

intersection_of: PATO:0001163 ! decreased concentration

intersection_of: qualifier PATO:0000460 ! abnormal

intersection_of: towards CHEBI:17234 ! glucose

intersection_of: inheres_in FMA:9670 ! Portion of blood

*Hypoglycemia *refers to a decreased concentration of glucose in the blood. The logical definition uses relations and follows the pattern described in previous work on defining phenotypes [4]. The logical semantics are made explicit in the translation to the Ontology Web Language (OWL) [15]. The translation used in this manuscript represents a relatively simple design pattern that nonetheless leads to the desired inferences.

Class: Hypoglycemia

EquivalentTo: 'decreased concentration' and towards some 'glucose '

and inheres_in some 'portion of blood' and qualifier some 'abnormal'

Note that we use the term labels rather than identifiers for the purposes of readability. Here, the class *Hypoglycemia *is defined as being equivalent to the intersection of all classes of things that are "A concentration which is lower relative to the normal" (*decreased concentration*), "deviate from the normal or average" (*abnormal*), with respect to (towards) glucose, and inhering in "blood" (using the term *portion of blood *from the Foundational Model of Anatomy (FMA) [16]). We use the formal inheres_in relation to relate qualities to their bearers - here the bearer of the quality is the blood. The relation towards is used to connect the quality (here, *decreased concentration*) to the additional entity type on which the quality depends (here *glucose*) [17]. We use this together with the term for *glucose *from the Chemical Entities of Biological Interest (ChEBI) ontology [18], essentially stating that the concentration is a concentration "of" glucose. We have thus defined *Hypoglycemia *as the intersection of these four classes. Defining ontology terms in this way assists in automating ontology construction, and provides a tool for integrative computational analysis of human and model organism phenotypes against the background of the knowledge incorporated in ontologies such as GO, FMA, and ChEBI [14].

In OWL, an ontology is a collection of axioms. An axiom can be thought of as a statement or a sentence, and includes ontological relationships such as those involving is_a or part_of. In the context of this paper, we say that axioms can be asserted (i.e. put there by the ontology curator) or inferred (deduced by a reasoner).

**Figure 1:**
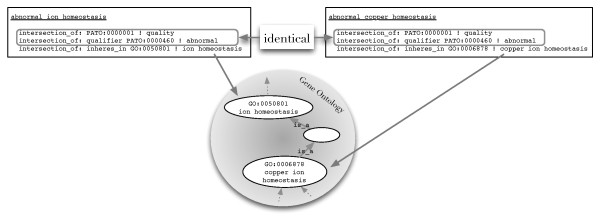
**Disagreement of ontologies**. In the MPO, *abnormal ion homeostasis *and *abnormal copper homeostasis *are not connected by an ancestor/descendent relation. The results of analysis by *GULO *of logical definitions of the MPO terms suggests that *abnormal copper homeostasis *should be a subclass of *abnormal ion homeostasis*, because the term *copper ion homeostasis *is a descendant of *ion homeostasis *in the GO. These two terms are used to logically define the corresponding terms in the MPO.

Having created logical definitions, one can apply automatic reasoners, which are systems for computing the logical consequences that can be inferred from a set of asserted axioms. Because reasoning systems can infer the positions of classes in a subsumption hierarchy based on their computable, logical definitions, they can serve as powerful tools in ontology development and maintenance [4]. The asserted subsumption hierarchy of a target ontology (such as the MPO) should be a logical consequence of the definitions of the terms. As seen in Figure 1 the terms *abnormal ion homeostasis *and *abnormal copper homeostasis *of the target ontology, the MPO, are defined logically. This has been done by referencing the GO term *ion homeostasis *for the first and *copper homeostasis *for the latter MPO term. Since there exists a subsumption axiom between the referenced GO terms (and the PATO terms used are identical) a reasoner will infer that the MPO term *abnormal copper homeostasis *is a subclass of the MPO term *abnormal ion homeostasis*.

Thus we assume that this relationship should also be asserted in the MPO, i.e., the knowledge represented in building block ontologies should generally be reflected in the target ontology as well.

We assume that a major goal is to create agreement between the relationships that are asserted in the target ontology and those that can be inferred from the building block ontologies. This can pave the way for extensive data integration with other ontologies to facilitate mining and querying biological knowledge [17]. The creation of the logical definitions for ontology terms mainly depends on manual curation by experts. The curators have to deal with imprecision, missing knowledge and regular changes and updates in the referenced ontologies, and so this can be an enormously complex task. Therefore, one approach to evaluate a target ontology is to run an automatic reasoner over the combined ontologies and logical definitions and then to check how well the manually asserted class-level axioms agree with the ones found by the reasoner. Depending on the knowledge and the kind of disagreement detected, one may either adapt the logical definitions or revise the subclass relationships of the target ontology. Of course cases may also exist in which the knowledge of the target ontology cannot be fully reasoned over, or cases in which the asserted relationships and inferred relationships represent different views or aspects of knowledge on a particular entity. In many cases, however, it is likely that a disagreement between the asserted hierarchy in ontologies such as the MPO or the HPO with the hierarchies of ontologies for anatomy, biological process, cell types, etc., indicates a logical error that should be manually reviewed by a curator. The developers of the logical definitions therefore need simple tools to evaluate their definitions and the target ontology in a fast and easy way.

Currently, the standard approach is to do minimal assertion in the target ontology, and using a reasoner such as Pellet [19] or HermiT [20] to deductively infer the target ontology polyhierarchy. Most ontology environments such as Protégé4 [21,22] or TopBraid Composer [23] are geared towards this workflow. However, we are faced with the reverse situation in application-driven biological ontologies where the target ontology is often constructed before the building block ontologies, and we want to leverage assertions in the target ontology to improve the building block ontologies and the logical definitions through an iterative process of mutual alignment. This has been the case for both the Gene Ontology logical definitions [4] and for phenotypes [17]. For example, the MPO frequently classifies phenotypes anatomically, which when combined with logical definitions allows us to improve anatomical ontologies. Existing OWL-based deductive reasoning environments are less well suited to this "two-way" information flow scenario [24]. In addition, reasoners can have unpredictable performance when used with multiple large ontologies such as the FMA.

Numerous other schemes have been put forward to increase coverage, consistency and quality of biomedical ontologies. These include graph-based approaches [25], linguistic methods for improvement of term names [26], and others (e.g. [27]). A related approach [25] runs only in conjunction with Protégé Frames. The lack of a freely available tool that is based on OBO and OWL semantics and is able to work with more complex logical definitions motivated the work presented here.

## Implementation

In this work, we present and implement a method for using automated reasoning to evaluate a set of logical definitions against the target ontology compared with the knowledge represented by all of the ontologies referenced in the logical definitions. The method first collects only the parts of the referenced ontologies that are relevant for reasoning (Table 1). Note that the referenced ontologies are expected to be provided with subsumption axioms. For the ontologies being considered in this work, it is only necessary to import terms that are directly referenced in the logical definitions and all of their ancestors back to the root in order to infer subclass relationships in the target ontology (Figure 1). We will refer to the graph made up of all referenced terms on an ontology as well as all ancestors on all paths back to the root as the *induced ancestral graph *of the ontology. Note that we add all relationships between the extracted terms to this graph. For example, by looking at the definitions of the terms from the MPO [28] the induced ancestral graph contains only 1,528 classes of the 35,000 classes in the complete GO (Table 1). Since reasoning does not require any of the other terms in the referenced ontology, one can obtain identical reasoning results over a smaller ontology containing just these terms with a substantial savings in computational resources. There are three types of disagreements between the logical definitions and the target ontology that we would like to detect:

**Table 1:** 10 External ontologies used for the MPO test-run

*Ontology*	*Number classes in Ontology*	*Fraction of terms referenced in logical definitions*
Chemical Entities of Biological Interest	44,843	2.71 %
Gene Ontology	35,090	4.35 %
Protein Ontology	26,727	0.18 %
Molecule Role Ontology	9,530	0.66 %
Uber-Anatomy (UBERON)	8,111	17.70 %
BRENDA Tissue Ontology	4,975	0.28 %
Adult Mouse Anatomical Dictionary	2,994	43.05 %
Phenotype, Attribute and Trait Ontology	2,283	23.87 %
Cell Ontology	1,510	30.20 %
Mouse Pathology	643	18.97 %

*Total*	140,453	7.45 %

Less than 10 % of the terms of the external ontologies are used by the logical definitions of the MPO terms. *"Fraction of terms referenced in logical definitions" *denotes the percentage of terms in the ontology that are members of the induced ancestral graph and are used by the logical definitions of the MPO terms.

1. A subclass relationship (an is_a link) is implied by the logical definitions but is not explicitly asserted in the target ontology.

2. A subclass relationship (an is_a link) is asserted in the target ontology but is not implied by the logical definitions.

3. The logical definitions imply that two separate classes (terms) in the target ontology are in fact equivalent.

While these three kinds of disagreements are trivial to detect in OWL ontologies, current software such as Protégé was not designed to present lists of detected disagreements in a way that curators can easily use for ontology maintenance. The software presented in this work, *GULO *(Getting an Understanding of LOgical definitions; Taxonomic note: *Gulo gulo*, the wolverine, notably includes owls in its diet), therefore imports the induced ancestral graphs from all ontologies referenced in the logical definitions of the the target ontology, uses computational reasoning to identify the set of all relationships between terms of the target ontology that are implied by the logical definitions and the referred ontologies, and compares them to the relationships that have actually been asserted in the target ontology. Any disagreement is then presented to the user in a set of easy-to-use files together with the reasons derived by the reasoner for the disagreement derived by the reasoner. These results can be used by ontology curators for ontology maintenance and debugging.

**Figure 2:**
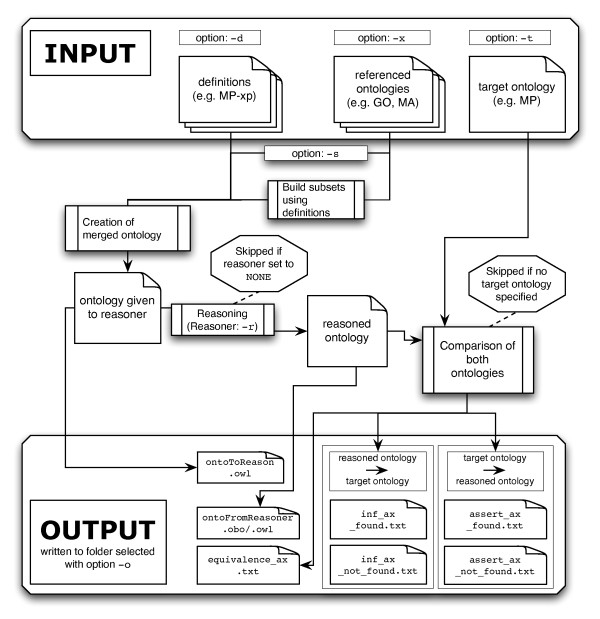
**Workflow and options of GULO**. A schematic representation of the workflow of *GULO *and how the user-specified options affect the workflow. Also files read, created and written are depicted.

We provide a stand-alone software implemented in Java that parses a set of definition files (the cross-product logical definitions) and a set of user-defined external ontologies that are referenced in the logical definitions. We will now explain the workflow together with the impact of the several program options that the user can specify. A schematic summary of the workflow is shown in Figure 2.

### Generation of relevant ontology subsets and running the reasoner

All the ontology files (definitions, external ontologies, target ontology) are parsed using the OWL API [29]. Note that for ontologies in OBO format we use the oboformat library [30], which provides a parser for OBO format 1.4 syntax and an implementation of a mapping to OWL using the OWL API. We also use the OWLTools package [31], a convenience Java API on top of the OWL API, which among other things provides convenient graph-operation capabilities over ontologies.

After parsing the definition file(s) and the external ontologies, a single composite ontology is constructed. By default, the composite ontology is generated using only subsets (the induced ancestral graphs) of the external ontologies; alternatively, the external ontologies are imported in their entirety if the option -s is chosen. The composite ontology (Figure 2: "ontology given to reasoner") is written to file and if desired can be imported into Protégé for manual inspection. Following this, the reasoner is started. The reasoners FaCT++ [32], HermiT [20], or Pellet [19] can be specified using the option -r. Note that in order to use FaCT++, the corresponding FaCT++ Java Native Interface library must be defined and available in the Java library path.

### Comparison against target ontology

After reasoning, a new ontology is created from the inferred axioms (Figure 2: "reasoned ontology"). This ontology is then compared against the specified target-ontology (-t). *GULO *performs a comparison in both directions; i.e., the reasoned axioms are checked for presence in the target ontology ("Reasoned ontology → Target ontology") and conversely all links between (defined) terms in the target ontology are sought in the reasoned ontology ("Target ontology → Reasoned ontology").

#### Output files

*GULO *writes several files to a user-defined output folder (option -o). A list of the most important files that are generated by *GULO *are given in Figure 2 (Output). The merged ontology and the ontology generated by the reasoner are written to the output folder. During the comparison of these two ontologies the axioms that are found by the reasoner are written to inf_ax_found.txt/inf_ax_not_found.txt according to whether they are present/not present in the specified target ontology. Similarly we report every link in the target ontology between decomposed terms that the reasoner has detected/not detected in the file assert_ax_found.txt/assert_ax_not_found.txt

Note that inf_ax_not_found.txt and assert_ax_not_found.txt also list the definitions (which give the users hints for debugging) for all the listed axioms. Furthermore, a file with inferred equivalence axioms (equivalence_ax.txt) is generated, where the listed equivalences can either point to duplicates in the target ontology (in case of true equivalencies) or to errors in the logical definitions (i.e., when a curator has accidentally used the same definitions for non-equivalent terms).

## Results and Discussion

### Software for automated reasoning evaluations

Here we present an application that automatically reasons over a set of logically decomposed terms and evaluates the resulting ontology against a specified target ontology. The goal of this process is to define terms of a target ontology and use a reasoner to create a new ontology by only using the logical definitions. The resulting ontology ideally has the identical structure as the target ontology in which the subclass axioms have been manually asserted. Our program *GULO *generates subsets of the external ontologies that only contain the terms of the induced ancestral graphs that are referenced in the logical definitions.

Finally, mismatches between the manually asserted subclass axioms and the reasoned axioms are reported and can be used to improve the structure of the target ontology or the axioms used to define the terms contained in the target ontology.

### The Mammalian Phenotype Ontology

To demonstrate that our software is applicable and important for developers and curators of ontologies and/or logical definitions, we tested it by using the *Mammalian Phenotype Ontology *(MPO) and the corresponding definition file containing the logical definition of a majority of the MPO terms. Note that for our testing procedure we also generated a bridge between anatomical terms and UBERON [33], since the MPO logical definitions refer to ontologies that are cross-referenced by UBERON (e.g. MA and FMA). A bridging file was generated that transforms the subclass axioms of UBERON (e.g., MA cochlear duct is a subclass of the generic cochlear duct) into correspoding equivalence axioms (e.g., MA coclear duct is defined to be equivalent to the generic cochlear duct in UBERON). This step makes the output of GULO easier to interpret. The code and a runnable jar (GenerateUberonBridge.jar) can be obtained from the *GULO *subversion repository (see section *Availability and requirements*). The ontologies used and the output files are also listed there (see folder dataUsed_gulopaper/).

#### Running time and memory

For testing the computational requirements of our program, we also used the test case of the MPO and the corresponding definitions and ran *GULO *on a computer with an Intel Core 2 Duo (2.66 GHz) and 4 GB RAM. When using subset creation (Table 1) the reasoning (using HermiT) and evaluation took ~115 seconds (run with the VM-option -Xmx500M). Running the same analysis without the construction of subsets gave us identical reasoning/evaluation results, but took around 310 seconds (with the VM option-Xmx1100M).

#### Evaluating the MPO

As described above, *GULO *can be used to detect disagreements or differences in the representation of knowledge between an ontology created from automatically reasoned axioms using logical definitions and a target ontology (MPO), where the axioms between terms have been generated by manual curation (Table 2). The first file presents examples where reasoned axioms could not be found in the MPO (inf_ax_not_found.txt).

**Table 2:** GULO results

*Inferred axioms referring to the MPO (4,216)*	
- asserted in XP	19

- not asserted in XP	4,198

*Reasoned ontology *→ *Target ontology (MPO)*	

- number inferred axioms directly found	2,662

- number inferred axioms indirectly found	557

- number new axioms	997

*Target ontology (MPO) *→ *Reasoned ontology*	

- number asserted axioms found	2,662

- number asserted axioms not found	4,095

Statistics of *GULO *running on the MPO. A total of 4,216 axioms were inferred by GULO, of which 19 were directly asserted in the logical (XP) definitions. Of these, 2,662 were explicitly and 557 implicitly asserted by the MPO. 997 new axioms were identified that require attention by curators.

In the first example the reasoner infers that *abnormal copper homeostasis *(MP_0003951) is a subclass of *abnormal ion homeostasis *(MP_0001765). This inference originates from the knowledge representation in the GO, where *copper ion homeostasis *is a descendant of *ion homeostasis *(see Figure 1). In the MPO these two terms are currently not in any ancestor-descendant relation, which suggests that further manual curation of these terms is necessary.

A similar example is the reasoned subclass axiom *enlarged inguinal lymph nodes *(MP_0009623) and *enlarged lymph nodes *(MP_0000702). As before there is a disagreement between knowledge representation in the MPO and an external ontology (MA). In this case the MA ontology states that *inguinal lymph node *is a subclass of *lymph node*, an axiom that is not represented in the MPO. As before we suggest structural changes in the MPO, so that this part of the MPO is aligned with the corresponding part of the MA. Further examples of links suggested by *GULO *for addition to the MPO are *increased prostaglandin level *subclass of *increased unsaturated fatty acid level*, *decreased quadriceps weight *subclass of *decreased muscle weight*, and *long radius *subclass of *increased length of long bones*.

Note that in total *GULO *finds 997 subclass axioms between MPO terms that are currently not explicitly asserted in the MPO. These terms and the relationships between them now represent priority items for further manual curation.

#### Evaluating MPO logical definitions

Another important feature of *GULO *is that it can be used to identify curator errors made during the creation of logical definitions. We detected numerous disagreements, but here we will describe only one example. The file equivalence_ax.txt showed us an equivalence between *abnormal urine uric acid level *and *abnormal blood uric acid level *owing to an erroneous definition of *abnormal urine uric acid level *which used the MA term for *blood *instead of the term for *urine*.

*GULO *is capable of indicating more complex disagreements. An axiom generated by the reasoner (assert_ax_not_found.txt) is the subsumption axiom between *abnormal sperm motility *(MP_0002674) and *abnormal locomotor activity *(MP_0001392). This axiom is not contained in the MPO. Both terms were decomposed as *abnormal *(PATO_0000460) and *quality *(PATO_0000001). The disagreement here comes from the third term used to define both MPO terms, which is in the first case *sperm motility *(GO_0030317) and *locomotion *(GO_0040011) for the definition of the latter MPO term. This produces a mixture between a statement about the motility of a single cell (sperm cell) and about the movement behavior of a whole organism (here the mouse). Here we are not confronted with a curator error, rather GO is too unspecific, as can be seen in the definition of *locomotion *("*Self-propelled movement of a cell or organism from one location to another*"). It is hard to say what the best solution to this problem might be. One solution would be for GO to restructure terms representing movement such that cellular movement and the movement of an entire organism are represented by separate hierarchies of terms.

## Conclusions

We provide a software package (*GULO*) for automatic reasoning over a set of logical definitions and the ontologies referenced by the definition statements. The referenced ontologies are automatically reduced by removing all classes that are not referred to by the definitions in order to reduce computation time and memory requirements. We assume that the ontology generated by the reasoner optimally should reflect the structure of the manually asserted links given in the target ontology. The reasoned ontology and the target ontology are compared with each other and disagreements are listed. These lists of differences are a powerful resource for the detection of errors in both the logical definition statements and the structure of the target ontology. Of course there may also be cases in which the knowledge of the target ontology cannot be fully reasoned over or cases in which the asserted axioms and inferred axioms represent different views or aspects of knowledge on a particular entity. The methodology presented here can thus be used as a system to help expert curators efficiently identify terms and relationships that require attention. The method is not intended to be used to automatically repair or generate an ontology.

The software presented here uses standard techniques for reasoning over OWL DL ontologies. It is especially designed to be used by curators of biomedical ontologies that use logical, cross-product definitions [4] for the classes of the ontology. This is currently the case for several prominent ontologies in the OBO Language, including the GO, the MPO, the HPO, and the Worm Phenotype Ontology [9]. We have demonstrated the usage of *GULO *by applying it to the manually created logical definitions of the terms of the MPO. We explained in which way users can get hints for disagreements and errors in both the MPO and the corresponding logical definitions of MPO terms. Curators of logical definitions of any kind of biomedical ontologies can use *GULO *as a tool for validation and consistency checking.

## Availability and requirements

• Project name: *GULO *(Getting an Understanding of LOgical definitions)

• Project home page: http://compbio.charite.de/svn/hpo/trunk/src/tools/gulo

• Operating system(s): Platform-independent

• Programming language: Java

• Other requirements: Java 1.5 or higher

• License: New BSD License

## List of abbreviations

MP/MPO: Mammalian Phenotype (Ontology); HPO: Human Phenotype Ontology; GO: Gene Ontology; MA: Mouse Anatomy; PATO: Phenotype, Attribute and Trait Ontology

## Competing interests

The authors declare that they have no competing interests.

## Authors' contributions

SK, SB, CJM, PNR, PS, and GVG planned the research work and set up the experiments. SK, SB, and CJM implemented the software. SK, GVG, PS, GC, and CLS performed the evaluations. SK, SB, CJM, and PNR contributed to writing the manuscript. All authors have read and approved the final version of the manuscript.
